# Multi-Valvular Non-bacterial Thrombotic Endocarditis Causing Sequential Pulmonary Embolism, Myocardial Infarction, and Stroke: A Case Report and Literature Review

**DOI:** 10.7759/cureus.32261

**Published:** 2022-12-06

**Authors:** Mutaz Karameh, Mordechai Golomb, Ariela Arad, Guy Kalmnovich, Eyal Herzog

**Affiliations:** 1 Heart Institute, Hadassah University Medical Center, Faculty of Medicine, Hebrew University of Jerusalem, Jerusalem, ISR; 2 Hematology Department, Hadassah University Medical Center, Faculty of Medicine, Hebrew University of Jerusalem, Jerusalem, ISR; 3 Sharett Institute of Oncology, Hadassah University Medical Center, Faculty of Medicine, Hebrew University of Jerusalem, Jerusalem, ISR

**Keywords:** marantic endocarditis, trousseau’s syndrome, stroke, pulmonary embolism, hypercoagulability of malignancy, percutaneous aspiration thrombectomy, coronary embolism, non-bacterial thrombotic endocarditis, acute myocardial infarction

## Abstract

Non-bacterial thrombotic endocarditis is an uncommon entity that tends to be related to malignancy or rheumatological disorders. The diagnosis is complex and requires a high index of suspicion. It commonly causes recurrent emboli; however, coronary embolism remains an infrequently reported entity. Herein we report a unique case of sequential pulmonary embolism, ST-elevation myocardial infarction (MI), and stroke associated with multi-valvular non-bacterial thrombotic endocarditis. The cornerstone of management is treating the underlying cause and anticoagulation therapy. Surgical treatment should be considered in patients with acute heart failure secondary to valvular dysfunction and recurrent thromboembolism despite proper anticoagulation. We have performed an extensive literature search and found nine cases of established antemortem diagnosis of myocardial infarction secondary to non-bacterial thrombotic endocarditis, and we reviewed them according to cause, treatment, and outcome.

## Introduction

Cancer is a known cause of hypercoagulability, which can present clinically in various ways, including migratory superficial thrombophlebitis (Trousseau's syndrome), superficial and deep venous thromboembolism, arterial thrombosis, disseminated intravascular coagulation (DIC), and non-bacterial thrombotic endocarditis.

Non-bacterial thrombotic endocarditis (NBTE) is a rare entity and tends to be related to malignancy or rheumatologic disorders [1]. NBTE is vegetation on cardiac structures, particularly the valves, consisting of fibrin and platelet aggregates without the presence of an infectious agent. We report a rare case of multivalvular non-bacterial thrombotic endocarditis complicated with pulmonary embolism, acute ST-segment elevation myocardial infarction (MI), and stroke. We also reviewed cases of established antemortem diagnosis of myocardial infarction secondary to non-bacterial thrombotic endocarditis according to cause, treatment, and outcome.

## Case presentation

A 47-year-old woman with a prior history of smoking and psoriasis treated with Enbrel 50 mg once a week but otherwise healthy presented in July 2021 to our health care center with deep venous thrombosis and pulmonary embolism (PE). Anticoagulation therapy was started. Her workup revealed lung adenocarcinoma in the left upper lobe with metastasis to the left hilum, bilateral mediastinal lymph node, and suspected spread to both lungs with no distant metastasis. Due to the impression of a significant illness, she got one cycle of chemotherapy (Cisplatin & Pemetrexed). Later, next-generation sequencing (NGS) testing showed a common pathogenic mutation in epidermal growth factor receptor (EGFR) exon 21 (L858R) and high PLD1. The treatment was changed (according to national guidelines) to targeted therapy with 3rd generation EGFR inhibitor osimertinib at a dose of 80 mg per day.

**Figure 1:**
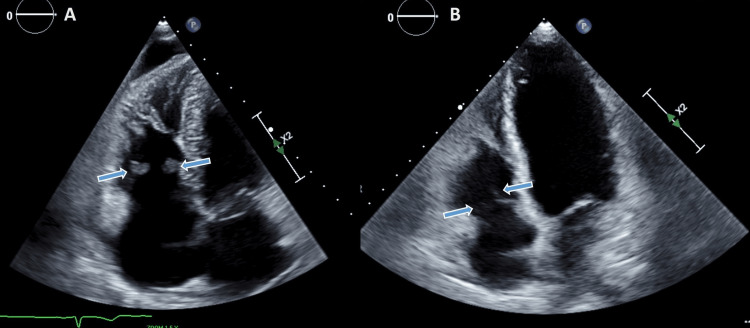
Transthoracic echocardiography (A) 4-chambers view showing vegetations on the septal and anterior leaflet of the tricuspid valve (blue arrows). (B) Same view after three months demonstrating the disappearance of the vegetations.

Four months later, in December 2021, she was readmitted again with new PE and vaginal bleeding despite continued anticoagulation. Her laboratory tests demonstrated disseminated intravascular coagulation (DIC). Transthoracic echocardiogram (TTE) demonstrated vegetation on the septal and the anterior leaflets of the tricuspid valve with severe tricuspid regurgitation (TR) and severe pulmonary hypertension (Figure 1A). IVC filter was inserted. Due to the worsening of her clinical condition with a manifestation of a coagulation disorder, her biological treatment was discontinued, and she was started again on chemotherapy regimen with carboplatin and paclitaxel along with continued treatment of the DIC. Within three weeks, she showed clinical improvement and resolution in terms of DIC parameters. Three months later, TTE demonstrated the disappearance of the vegetation with mild TR and resolution of pulmonary hypertension (Figure 1B). Laboratory workup excluded signs of DIC.

**Figure 2:**
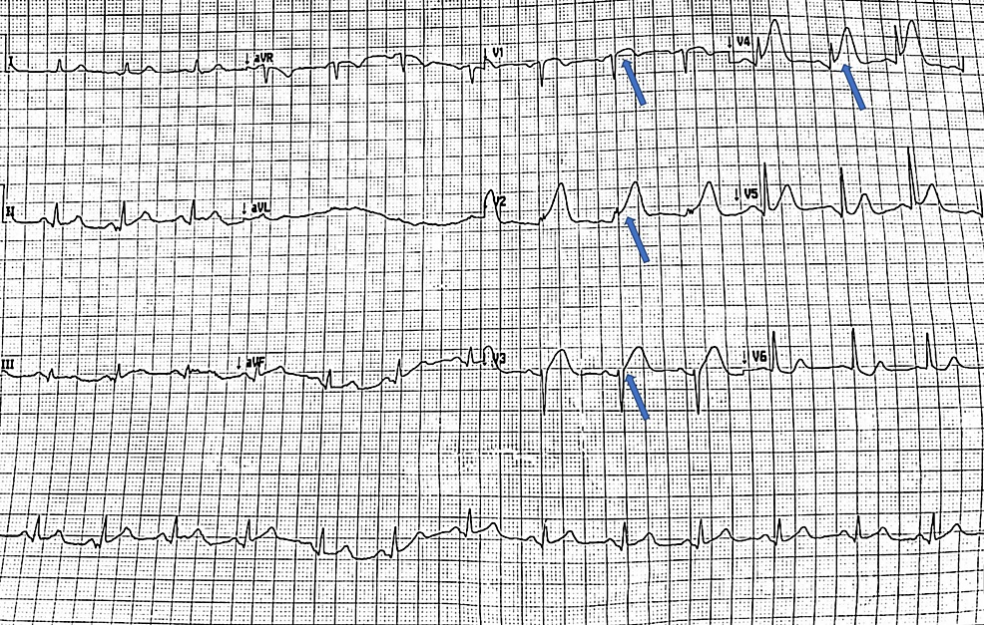
Electrocardiogram Electrocardiogram showing sinus rhythm with ST-segment elevation in anterior precordial leads (arrows).

**Figure 3:**
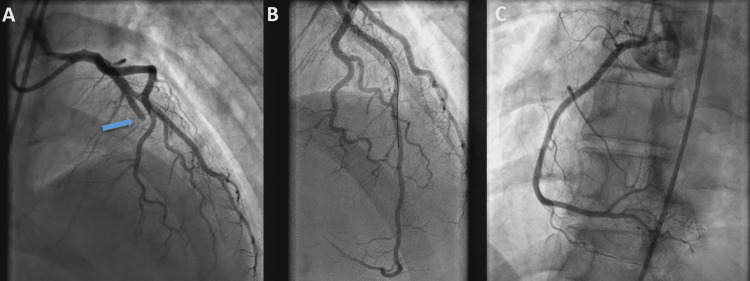
Coronary angiography Abrupt occlusion of the middle part of the left anterior descending artery (arrow) causing ST-elevation myocardial infarction (A) with complete revascularization after percutaneous coronary intervention (B). (C) Right coronary angiogram showing a normal artery.

In May 2022, the patient presented with worsening leg pain, and laboratory tests again showed evidence of DIC. She was readmitted for evaluation. Two days later, an electrocardiogram (ECG) was performed due to acute-onset chest pain and showed sinus rhythm with anterior ST-segment elevation myocardial infarction (Figure 2). The patient rapidly deteriorated to ventricular fibrillation. She was successfully resuscitated with multiple defibrillator shocks. She was intubated and spontaneous circulation was restored. She was rushed to the cardiac catheterization laboratory, where an urgent coronary angiogram was performed and revealed abrupt occlusion of the proximal part of the left anterior descending artery. She was treated successfully with the implantation of a drug-eluting stent (Video 1) (Figure 3).

**Video 1:** Coronary angiogram Injection to the left coronary system in the cranial right anterior oblique (RAO) view showing abrupt occlusion of the left anterior descending artery with otherwise normal arteries.

**Figure 4:**
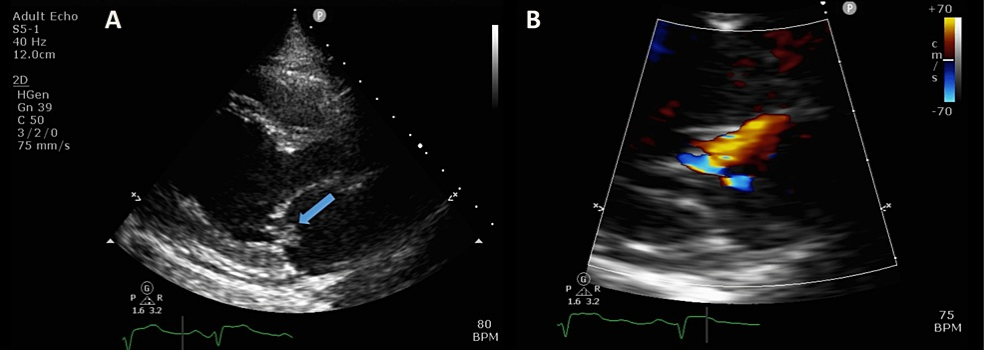
Transthoracic echocardiography (A) Small mass seen on the posterior leaflet of the mitral valve (white arrow) in parasternal long-axis view. (B) Color Doppler echocardiogram in the same view with an eccentric jet of severe mitral regurgitation.

**Figure 5:**
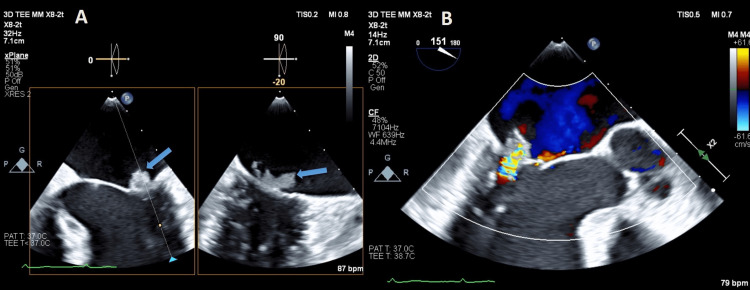
Transesophageal echocardiogram (A) Vegetation on the posterior mitral leaflet (arrow) and with an X-Plane view. (B) Color Doppler transesophageal echocardiogram showing severe mitral regurgitation.

**Figure 6:**
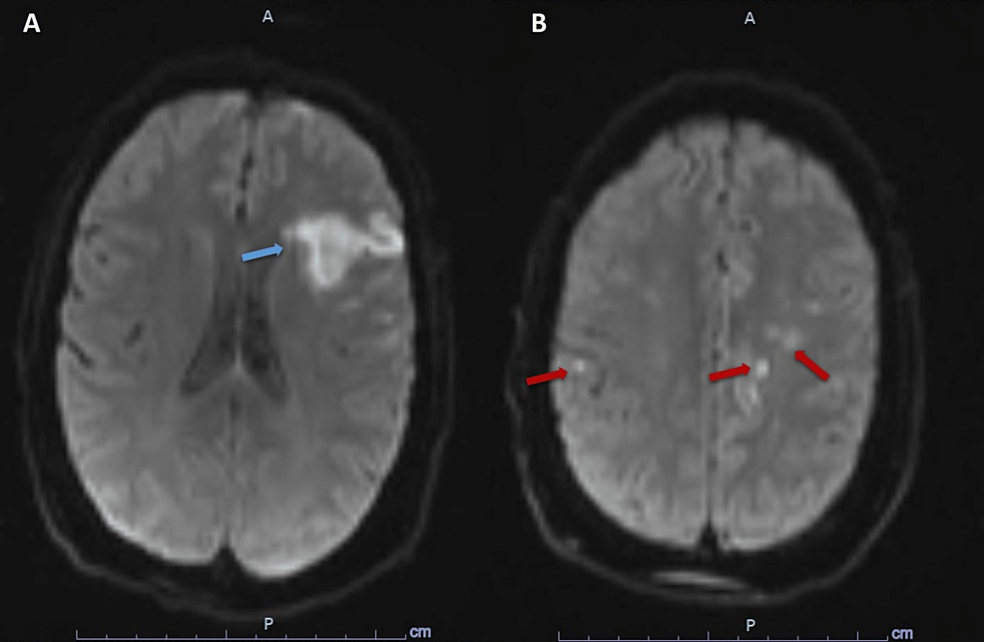
Magnetic resonance imaging (A) Diffusion magnetic resonance imaging demonstrating infarct areas in the left frontoparietal lobe (Blue arrow). (B) Patchy infarct areas in the left and right parietal lobes (red arrow).

**Figure 7:**
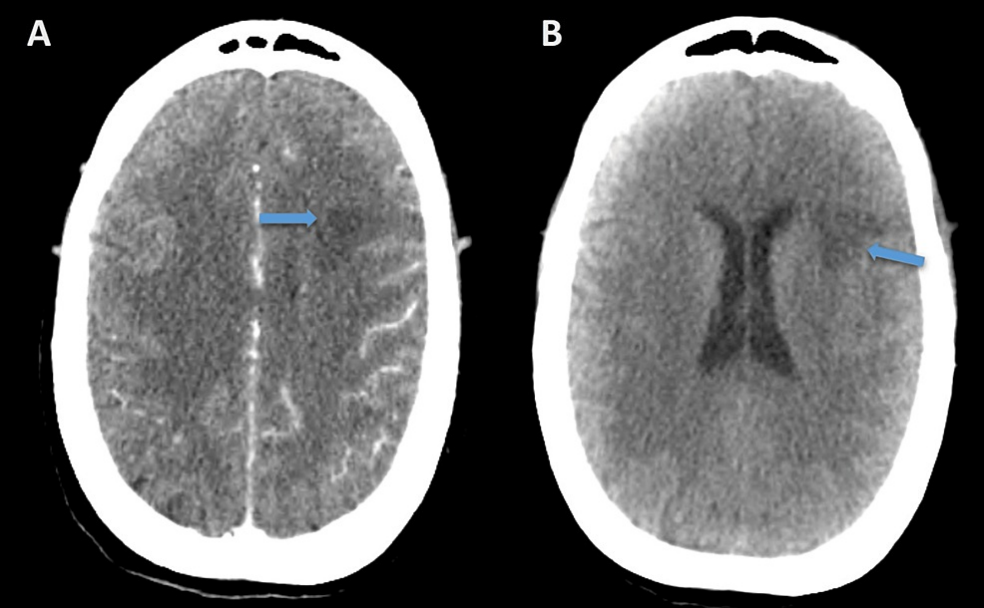
Computed tomography The transverse view that demonstrates hypodense areas of encephalomalacia consistence of infarction (arrows) with (A) and without (B) contrast media.

The patient was admitted to the cardiac intensive care unit on mechanical respiratory support. Transthoracic echocardiogram (TTE) showed a severely decreased left ventricular ejection fraction (LVEF), a large eccentric jet of mitral regurgitation (MR), and a small oscillating mass on the posterior leaflet of the mitral valve (Figure 4). There was no evidence of intracardiac thrombus, and there was no evidence of intracardiac shunt after agitated saline injection, excluding paradoxical emboli. Subsequent transesophageal echocardiography (TEE) confirmed severe MR and showed an independently oscillating mobile vegetation measuring 10x12x5 mm (Figure 5) (Video 2) and normal tricuspid valve. No thrombus was seen in the left atrial appendage. Due to her general condition and hemodynamic instability, she was not a candidate for surgical intervention after a multidisciplinary discussion. After weaning from sedation, she woke up with notable right hemiparesis and aphasia. Computed tomography and magnetic resonance imaging (MRI) revealed signs of acute infarctions in the left frontoparietal and right parietal hemispheres, more prominent on the left side, secondary to an embolic event (Figures 6, 7).

**Video 2:** Transesophageal echocardiogram Mid-esophageal view at 0 degrees and X-plane showing the mitral valve with a notable mobile mass attached to posterior leaflet consistent with vegetation.

She was empirically treated with antibiotic therapy with a diagnosis of aspiration pneumonia and suspected infective endocarditis (IE).

Physical examination and laboratory tests revealed no peripheral signs of infective endocarditis, and the patient remained afebrile throughout her hospitalization. Blood cultures were sterile, including two sets drawn before antibiotics administration. Throughout her admission, the patient gradually improved neurologically and thus was transferred to the oncology ward.

Unfortunately, her clinical condition deteriorated over the next few days. She developed DIC followed by acute respiratory distress requiring intubation and mechanical ventilation. She went into asystolic cardiac arrest and despite prolonged resuscitation efforts, she passed away with her family at her side. PE or recurrent coronary embolism were thought to be the mechanisms involved in her deterioration.

## Discussion

Malignancy is a known cause of hypercoagulability, which can present clinically in various ways, including migratory superficial thrombophlebitis (Trousseau's syndrome), superficial and deep venous thromboembolism, arterial thrombosis, DIC, and non-bacterial thrombotic endocarditis (NBTE).

NBTE is a rare condition and tends to be related to malignancy or rheumatologic and autoimmune disorders such as systemic lupus erythematosus [1]. NBTE is a vegetation on cardiac structures, particularly the valves, consisting of fibrin and platelet aggregates without the presence of an infectious agent [1]. Zeigler described the deposition of fibrin on cardiac valves and called it thromboendocarditis in 1888 [2]. In 1936, Gross and Friedberg first used the term 'non-bacterial thrombotic endocarditis' [3].

Recurrent emboli are common in both IE and NBTE, occurring in up to 50% of patients and typically affecting the central nervous system [2]. Despite frequent systemic embolic phenomena, coronary embolism remains an infrequently reported entity.

In our patient, factors suggesting an embolic source for her coronary event include the lack of atherosclerotic plaque and collateral vessels and the abrupt occlusion of the artery, without evidence of a spontaneous dissection (a common cause of MI in young females), while the other coronary arteries were angiographically normal.

The pathogenesis of NBTE is not fully understood; several mechanisms play a role. The primary factor is local damage mediated by elevated circulating cytokines, a malignancy-associated hypercoagulable state, and subsequent platelet aggregation on subendothelial connective tissue. The preexisting valvular lesion, if present, significantly further enhances this process [1].

Roux et al. [4] in the largest case series of 1210 patients with infective endocarditis, reported that 2.2% developed an acute coronary syndrome (ACS). ACS is mainly caused by coronary embolism and, to less extent, mechanical compression of the vegetation. Coronary embolisms are more frequently observed in patients with aortic valve involvement, most probably due to the proximity between the aortic vegetations and coronary Ostia [4]. To our knowledge, robust data on ACS in NBTE is unavailable.

NBTE should be considered in all cases of DIC and malignancy with systemic emboli. Once suspected, TTE should be performed. The cornerstone of the management of NBTE is treating the underlying cause. Initiation of empirical antibiotic treatment after obtaining blood cultures should be considered. Anticoagulation is warranted if there are no contraindications [1]. Low molecular weight heparin is the suggested treatment for NBTE in the American College of Chest Physicians guidelines [5]. In curable cancers, coagulopathy should be corrected, and a multidisciplinary approach regarding the need and timing of surgery should be considered. No consensus surgical guidelines exist in the management of NBTE. Surgery should be considered in patients with acute heart failure secondary to valvular dysfunction and patients with recurrent thromboembolism despite proper anticoagulation therapy. The decision of surgical intervention in stable patients without complications is complex and requires multidisciplinary discussion.

The patient described in our case was diagnosed with NBTE of the tricuspid valve and pulmonary embolism a few months before her current admission. She was successfully treated with anticoagulation and chemotherapy with the disappearance of the vegetation after three months.

Because of the low incidence of coronary embolism in both IE and NBTE, the data in the literature regarding its management are scarce. Primary percutaneous coronary intervention (PCI) should be performed in ST-segment elevation myocardial infarction (STEMI) secondary to NBTE. According to the 2017 European Society of Cardiology Clinical Practice Guidelines, routine thrombus aspiration in embolic STEMI is not recommended [6].

A review of the literature revealed many case reports of embolic myocardial infarction in autopsy or clinical circumstances, but only nine cases were found with an established antemortem diagnosis of acute myocardial infarction secondary to NBTE.

Table 1 summarizes these nine cases. Two out of nine patients presented with NSTEMI, and the others were with STEMI.

**Table 1:** Cases with established antemortem diagnosis of acute myocardial infarction secondary to non-bacterial thrombotic endocarditis. The table summarizes the cases of established antemortem diagnosis of acute myocardial infarction secondary to non-bacterial thrombotic endocarditis. STEMI: S-T segment elevation myocardial infarction, NSTEMI: non-S-T segment elevation myocardial infarction, M: male, F: female, LAD: left anterior descending artery, PLA: postero-lateral artery, PDA: posterior descending artery, RCA: right coronary artery, OM: obtuse marginal, APLA: antiphospholipid syndrome, RA: rheumatic arthritis, SLE: systemic lupus erythematosus.

Author	Age	Sex	Survival	Type	Artery	Valve affected	Intervention	Other systemic emboli	Underlying cause
Gray et al. [7]	66	M	No	NSTEMI	PLA and PDA	Mitral	Aspiration	No	Lung cancer
Trovato et al. [8]	29	M	Yes	STEMI	LAD	Aortic	Balloon	No	APLA
Garcia et al. [9]	46	F	Yes	STEMI	Distal PLA	Aortic	No intervention	Right anterior and posterior tibial arteries	RA
Patel and Elzweig [10]	66	F	No	NSTEMI	No culprit	Mitral and aortic	No intervention	Stroke	Pancreatic cancer
Kocabay et al. [11]	26	M	Yes	STEMI	Distal PDA and distal PLA	Mitral	Balloon	No	SLE
Boris et al. [12]	74	M	Yes	STEMI	Distal RCA	Mitral and aortic	Aspiration	Subclinical stroke	Biliary adenocarcinoma
Tiong et al. [13]	57	F	No	STEMI	Distal OM	Aortic	Aspiration	No	Cervical adenocarcinoma
Ferlan et al. [14]	43	F	Yes	STEMI	Distal PDA	Mitral	No intervention	Stroke	Gastric adenocarcinoma
Nagano et al. [15]	29	M	Yes	STEMI	Proximal LAD	Aortic	Aspiration and stent	No	Burger's syndrome

Six of these had survived. In three patients, the mitral valve was involved, in four the aortic valve was involved, and in two patients, both valves were affected. Three of them were treated with aspiration only, two with a balloon, one with stent implantation, and in three patients, no intervention was done due to the distal position of the lesion. In five patients, there was underlying malignancy; in the others, there was evidence of rheumatological diseases (systemic lupus erythematosus, rheumatoid arthritis, antiphospholipid syndrome, and Burger’s syndrome). In four patients, embolic stroke and other systemic emboli were reported. Surgical removal was done only in two patients.

## Conclusions

NBTE is a rare condition and requires a high index of suspicion. Herein we report a unique case of sequential pulmonary embolism, ST-elevation myocardial infarction, and stroke associated with multi-valvular NBTE. We describe an extensive literature review and found nine cases of established antemortem diagnosis of myocardial infarction secondary to NBTE. No established guidelines exist. Each situation requires a multidisciplinary approach to select the best treatment. Treatment of the underlying cause and anticoagulation are the cornerstones of the treatment.
